# Two New *Nepenthes* Species from the Philippines and an Emended Description of *Nepenthes ramos*

**DOI:** 10.3390/plants5020023

**Published:** 2016-05-06

**Authors:** Thomas Gronemeyer, Wally Suarez, Herman Nuytemans, Michael Calaramo, Andreas Wistuba, François S. Mey, Victor B. Amoroso

**Affiliations:** 1 Institute of Molecular Genetics and Cell Biology, Ulm University, 89081 Ulm, Germany; 2 0309 Purok 1C, Barangay Longos, Kalayaan, Laguna 4020, Philippines; 3 Grotstraat 68, B-2990 Wuustwezel, Belgium; 4 Ecotourism Park and Botanic Gardens, Northwestern University, Laoag City 2900, Philippines; 5 Friedhofweg 4, 88437 Maselheim, Germany; 6 3 Rue Frédéric Chopin, 59320 Haubourdin, France; 7 Center for Biodiversity Research and Extension in Mindanao (CEBREM), Central Mindanao University, University Town, Musuan 8710, Philippines

**Keywords:** carnivorous pitcher plants, *Nepenthes*, Ilocos Norte, Mount Hamiguitan, Philippines

## Abstract

With 50 species of the genus *Nepenthes* L. currently described from the Philippines, it is without doubt that the country, along with the islands of Sumatra (Indonesia) and Borneo (Indonesia, Malaysia, Brunei), should be considered the center of diversity of the genus. In this work, we describe two new species. One species, *N. aenigma* *sp. nov*., is from Ilocos Norte province on Luzon Island and has the—for *Nepenthes*—unusual ecological preference to grow in dense vegetation in deep shade. The other new species is from Mount Hamiguitan in Davao Oriental province on Mindanao Island. With this new entry, Mount Hamiguitan is now home to four endemic species (*N. peltata*, *N. micramphora*, *N. hamiguitanensis*, *N. justinae sp. nov*.). Furthermore, we provide an emended description of *N. ramos* based on field data. *Nepenthes kurata* is synonymized here with *N. ramos*.

## 1. Introduction

The past few years have seen the description of several new *Nepenthes* species from the Philippines, thanks largely to expeditions undertaken in the last decade ([1,2] and references therein). Most of the species described were from the islands of Palawan and Mindanao, which perhaps reflected how little was known about the *Nepenthes* flora of the Philippine archipelago. The other large island, Luzon, has mainly been passed over, first and foremost due to the long-standing belief that only two species exist there viz. *N. alata* and *N. ventricosa*, despite the presence of many habitats and substrate types suitable for *Nepenthes*. However, recent years have witnessed a number of new species descriptions, most of them exclusively based on herbarium material collected over a span of several decades [3,4,5,6,7,8,9,10]. Importantly, the taxonomic concept of *N. alata* was revised. Based on this concept, *N. alata* is a pubescent species with one-flowered partial peduncles, which is restricted to Luzon. The resurrected *N. graciliflora* is the glabrous counterpart with a wider distribution [3].

In 2013, Martin Cheek and Matthew Jebb described *N. alzapan* from material collected by Maximo Ramos and Gregorio Edaño on Mt. Alzapan, Tayabas (now Quirino) in June 1925 [4], and *N. ultra* from specimens collected by Ridsdale in Zambales in 1986 [5]. Furthermore, they resurrected *N. blancoi* as *N. abalata* [6]. While the herbarium material of *N. alzapan* and the recently described *N. barcelonae* [7] display clear characteristics enabling them to be recognized as distinct species, several new taxa from the Visayas and particularly Mindanao were diagnosed based on minor morphological characteristics (pitcher shape, lid shape and size, shape of the indumentum, *etc.*) that make identification in the field rather difficult. The description of *N. ramos* [8] is one of the most comprehensive of the new species on Mindanao, as the sheets contain both pitchers and flowers. *Nepenthes ramos* differs from *N. alata* and *N. graciliflora* in having two-flowered partial peduncles. Some other descriptions of species from Mindanao like those of *N. kurata*, *N. extincta* or *N. cid* are based on incomplete herbarium specimens lacking flowers or even characteristical pitchers that allow the identification of the respective species in the field. Intermediate pitchers that are only formed for a brief period of time (*N. extincta*) or (presumably) juvenile pitchers (*N. cid*) were considered instead [9,10]. The ecology of the respective localities was not assessed based on field data.

We provide in the herein presented work an emended description of *N. ramos*. Based on data recorded in the field, we show that *N. ramos* is a very variable species and synonymize *N. kurata* under it as the characteristics that were used to describe this species fell in the natural variability of *N. ramos*.

Furthermore, we describe two new species based on field research, namely *N. aenigma*, a new species from Luzon, and *N. justinae* from the world heritage site Mount Hamiguitan (Mindanao).

## 2. Results

### 2.1. A New Pitcher Plant Species from Luzon

#### 2.1.1. Taxonomic Description of *Nepenthes aenigma*

***Nepenthes aenigma*** Nuytemans, W. Suarez, Calaramo, *sp. nov.*

Type:

Philippines, Luzon, Ilocos Norte, 17.06.2009, *M.* *Calaramo,* holotype Calaramo2288 (female flower) (HNUL, isotype HNUL)

Additional material examined:

Calaramo2270 (male flower) (24.6.2011, *M. Calaramo*) (HNUL)

Diagnosis:

Differs from *Nepenthes ventricosa* Blanco in having cylindrical, winged and dimorphic pitchers, linear to elliptic lamina and 2-flowered partial peduncles (*N. ventricosa*: pitchers waisted at the middle, wings reduced to ridges, long and narrow lamina, inflorescense with 1-flowered partial peduncles).

*Nepenthes aenigma* was previously documented as an incompletely diagnosed taxon under the name *N. sp. “Luzon”* [11].

Description:

A terrestrial, climbing vine. S*tems* up to 5 m long, glabrous, terete to triangular in transection, 5 to 6 mm in diameter; internodes are up to 5 cm in length. There are dormant buds situated 3 to 5 mm above each leaf base. The vining stems produce short stems formed above the ground, which produce traps nested or buried in leaf litter. Aged stems eventually die off at the tips but the growing point is taken up by aerial shoots which sprout from the aforementioned dormant buds and which grow rapidly and produce traps. One of the photographs originally taken from these plants, taken at the time of the discovery of the species, clearly shows this behavior [11].

*Leaves* are sessile, linear to ensiform with rounded to acute apices. They are up to 19 cm long by 5.4 cm wide and with 3 to 4 nerves on either side of the midrib; bases are strongly decurrent to the stems where they form narrow wings.

*Lower pitchers* are unknown. *Intermediate pitchers* originate abruptly from the tendril, which is at the side or the rear of the pitcher. Tendrils are 35 cm long, uncoiled and approx. 2 mm in diameter. The intermediate pitchers are barrel-shaped though the profile is slightly more elongated on those that are not buried in leaf litter. They are entirely cylindrical in transection and without a hip. They are 11.7–15.9 cm high by 4.1–6 cm in diameter. Wings are 4.5 mm wide with fringes often branched and measuring up to 4.85 mm long. The pitcher opening is circular and oblique to an angle of about 40 degrees when viewed from the sides. The lid is 41–48 mm long by 37–41 mm wide and has a cordate base. Lid glands are oblong, without borders and arranged in a V-like manner. The peristome is rounded in transection, up to 7 mm wide from the front of the mouth to 8.5 mm in breadth near the lid. Beneath the lid, the peristome is appressed to form a short neck. Peristome ribs are 0.5 mm high and spaced 0.8 mm apart. Inner edges of ribs end in downward pointing teeth up to 0.8 mm long from those near the lid but shorter elsewhere. Outer peristome margins are curved and slightly crenellated. The spur is 2.5 mm long and bifid at the tips.

*Upper pitchers* originate rather abruptly from the tendrils but less so than those in intermediate pitchers; they are cylindrical and slightly curved forward with the bases widely infundibular; 7.1 to 11 cm high by about 3 cm wide near the pitcher mouth. The peristome is somewhat flattened, 4.7 mm wide and broadening to 6.2 mm near the lid. Peristome ribs are 0.5 mm high and spaced 0.7 mm apart. Inner margins bear barely discernible teeth; outer margins are only moderately curled and only sparsely crenellated. The pitcher opening is circular and oblique to 30 degrees from the horizontal. The lid is oblong with cordate base, hardly keeled beneath, lacks an appendage and is 29 mm long and 25 mm wide. Lid glands are more numerous near the lid base, crateriform but very tiny. Wings are 2.4 mm wide with filaments up to 2.9 mm long, end abruptly at ⅔ to ⅞ of the pitcher length and as such do not reach the pitcher mouth. Higher up, the wings are reduced to narrow ridges. Sometimes, these wings are not symmetrical, one wing being markedly longer than the other one on the same pitcher. The spur is simple and 1.8 mm long. Tendrils are twice coiled.

The *inflorescence* develops from the axils, sub-terminal on stem. The male inflorescences measure 15–30 cm with mostly two-flowered partial peduncles. It is pubescent with flowers 6–9 mm in diameter; the pedicel is 6–8 mm long. Four tepals are present with elliptic apex and acute bases to 5 mm long; staminal column 4–5 mm long. Female flowers have an elliptic corolla, with bases and apices acute. Four pistils are present with fused apices, being white in color. The infructescence is unknown.

*Indumentum* is not significantly present; the plant is mostly glabrous, but short (0.35 mm), brown hairs are found on undeveloped traps and their subtending tendrils. In opened pitchers, indumentum is confined to the spur.

The color of the stems is bright green to dark reddish; the leaves are green with green midribs adaxially, green to red abaxially. Intermediate pitchers are pale green with elongated vertical dark red spots that often coalesce into larger blotches and which are densest underneath the lid. Lids are pale green with numerous tiny red spots that are mostly distributed on the margins. The peristome is cream to reddish, less commonly dark red, on those traps that form on the ground but is green with dark red suffusion on aerial pitchers. Upper pitchers are entirely green. Tendrils of intermediate pitchers are colored red, and green on the upper traps.

**Figure 1:**
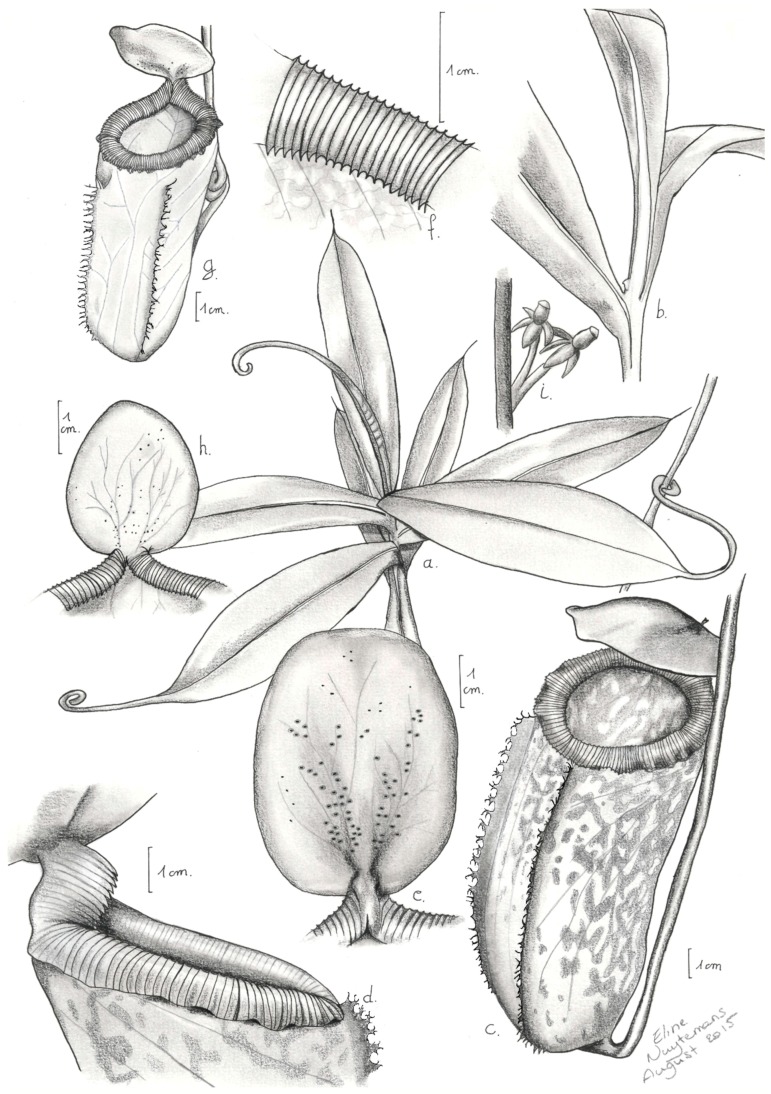
Botanical illustration of *N. aenigma*. (**a**) stem with leaves; (**b**) climbing stem with dormant bud; (**c**) intermediate pitcher; (**d**) column and outer rim peristome of intermediate pitcher; (**e**) lid of intermediate pitcher, lower surface; (**f**) inner edge of intermediate pitcher peristome; (**g**) upper pitcher; (**h**) lid of upper pitcher, lower surface; (**i**) female flowers. Drawing by Eline Nuytemans.

**Figure 2:**
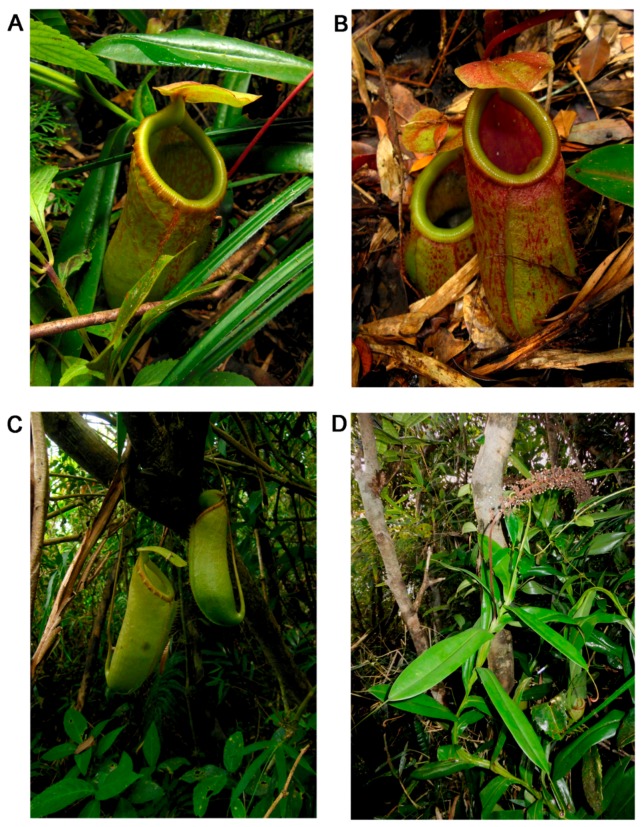
Photographs of *N. aenigma* at the type locality. (**A**, **B**) intermediate pitchers; (**C**) upper pitchers; (**D**) climbing stem with withered male inflorescence. Photos by Herman Nuytemans and Wally Suarez.

The morphological characteristics of *N. aenigma* and its related species are summarized in Table 1. Figure 1 shows a botanical illustration of *N. aenigma*. Photos of the species at the type locality are shown in Figure 2.

Etymology:

The specific epithet is derived from the Latin word *aenigma*, which means ”puzzling”, a reference to the very unusual ecological preferences of this new taxon. (see Section 2.1.2 and Section 3).

#### 2.1.2. Distribution and Ecology

*Nepenthes aenigma* is so far only known from three sites on two mountains in Ilocos Norte Province, island of Luzon. Site 1 and 2 are only some 100 m apart on the same mountain, with unfragmented surrounding vegetation. Site 3 is located on another mountain 10 km apart.

*Nepenthes aenigma* grows terrestrially in leaf litter at altitudes *ca.* 1200 m a.s.l. in deep shade in windswept ravines. The vegetation where the observed populations occur consists mostly of dense stands of bamboo, interspersed with various species of rattan and *Pandanus*. The vining plants have been seen to scramble and climb over these often spiny plants (Figure 2D), but were not seen to direct their growing tips and reach for brighter light. A mossy forest formation is absent despite the elevation in which this new *Nepenthes* occurs. This new taxon grows sympatrically with *N. ventricosa*, at least on Site 1, but separated by a narrow band of altitude, the latter often growing at slightly higher elevations. The plants from Site 2 have not been observed to grow sympatrically with other *Nepenthes* species. The single plant from Site 3 may be a representative of a larger population still awaiting discovery, and its flowering period is so far unknown. The mountain range in which these two species occur is quite extensive; the Northwesterniana Expeditions led by MC in the past years have tried to fill our gap in the knowledge of our plants in the area, but a sizable portion of the range remains little explored. Prey contents of the pitchers consisted mostly of ants, although unidentified spiders and roaches in advanced stages of decomposition were also noted. The traps also hosted unidentified mosquito larvae.

#### 2.1.3. Conservation Notes

Only five plants were observed on Site 1 in 2013, all of which were in their vining stages. The plants are extremely difficult to locate due to their preference for dense vegetation, and the two mountains on which they have been found are seldom climbed, even by locals or mountaineers. Even so, we are reluctant to reveal the name of the peaks as an added precaution against poaching.

All plants from Site 1 observed in 2015 were males, and no seedling plants were observed, even in 2013. This fact, coupled with the very low population density perhaps points to a natural extinction of this species on this site. Site 2 consisted of two very small populations, and Site 3 yielded a single plant.

Based from the very low number of plants found, it is easy to assess *N. aenigma* as “Critically Endangered” (CR) under IUCN [13], but it should be noted that all plants from the three known sites are not in any way threatened by poaching or habitat disturbances. Pending further population studies of this species, we recommend that *N. aenigma* be deemed “Data Deficient” (DD) with regards to its conservation status.

### 2.2. Another Endemic Species of Pitcher Plant from Mount Hamiguitan (Mindanao Island)

#### 2.2.1. Taxonomic Description of *Nepenthes justinae*

***Nepenthes justinae*** Gronem., Wistuba, Mey, V.B.Amoroso, *sp. nov*.

Type:

Philippines, Mindanao Island, Mount Hamiguitan, 17.08.2004, *V.B. Amoroso,* holotype
CMUH00003606 (CMUH).
Other material examined:
CMUH00003607 (CMUH) (17.08.2004, *V. B. Amoroso*) (Paratype)
ULM-22538, ULM-22539 (ULM) (18.2.2015, *T. Gronemeyer*, from a cultivated plant grown from seed).

Diagnosis:

Differs from *Nepenthes mindanaoensis* Sh.Kurata in having lower pitchers with a bulbous lower ⅔ and slightly infundibulate upper ⅓ (*N. mindanaoensis*: overall slender, cylindrical lower pitchers with a bulbous bottom). Lid of the upper pitchers with appendage (*N. mindanaoensis*: no appendage).

Description:

Th*e stem* is up to 4 m long, cylindrical in transection, 5–8 mm in diameter. The average internode length is 6 cm (might be longer in climbing stems).

*Leaves* of the climbing stem are elliptic to ovate, 18 cm long and 11 cm wide, with 3 equally distributed longitudinal veins on each side of the midrib and numerous pinnate veins running obliquely towards the leaf margins. The apex of the lamina is acute, the base attenuate and forming a sessile, narrowly winged petiole of 6 cm in length. Pitchers are on tendrils being up to 21 cm long. The tendrils are curled and are covered with short, brown hairs, about 1 mm long.

*Lower pitchers* are bulbous in the lower ⅔ and slightly infundibulate in the upper ⅓. They are 9 cm long and 2.5 cm wide in the upper half and 3.5 cm wide in the lower half. The pitcher narrows slightly towards the opening. Wings run down the front of the trap, fringed with filaments up to 3 mm long and 2 mm apart. The pitcher opening is distinctively oblique, up to 2 cm wide and acuminates towards the lid. The peristome is slightly flattened, up to 0.5 cm wide, not crenelated and densely lined with ribs having very small conspicuous teeth at the margin. The peristome is elongated into a neck towards the lid, which is cordate and 1.5 × 2 cm wide. Nectar glands are monomorphic and large with the largest glands located in a cluster distally to the midline, smaller glands cover approx. 60% of the lid surface. A keel, 3 mm wide, is present on the underside of the lid. The spur is 3 mm long and bifid at the tip but not completely branched.

The exterior of the lower pitchers is creamy-yellow, with narrow purple blotches. The interior of the pitcher is uniformly greenish-white and sometimes lined with angular blotches of purple. The peristome is dark red and unevenly yellow striped. The whole surface of the lower pitchers is covered with dense, very short brown hairs.

*Upper pitchers* are 17 cm long and 3.5 cm wide in the upper ⅔ and 4 cm wide in the lower ⅓. The lower ⅓ is separated by a distinct rim from the upper ⅔ of the pitcher. The pitcher narrows slightly towards the middle section before it widens towards the oblique pitcher opening; the latter being 2.5 cm wide. Wings run down the front of the trap, fringed with filaments up to 3 mm long and 4 mm apart. The wings are sometimes reduced to ribs. The peristome is slightly flattened, up to 1 cm wide, elongated into a short collar and densely lined with ribs having no visible teeth at the margin. The lid is cordate and 3 × 4 cm wide. A rounded appendage of 3 mm on a keel is present; the spur is 4 mm long, unbranched but is occasionally bifid at the tip. The nectar gland distribution, coloration and indumentum are consistent with the lower pitchers.

The *female* *inflorescence* is a panicle composed of a 40 cm-long scape and an additional 25 cm rachis. The partial peduncles are 2-flowered and 12 mm long. The pedicels are 10 mm long bearing flowers with 4 mm wide tepals. The male inflorescence is unknown.

*Note:* The given dimensions are based on the holotype specimen and of the specimens at ULM. The dimensions of the pitchers may be larger in all respects. At the type locality upper pitchers up to 25 cm large have been recorded. Dimensions of the female inflorescence were recorded from the specimen at ULM as the holotype does not contain inflorescences.

**Figure 3:**
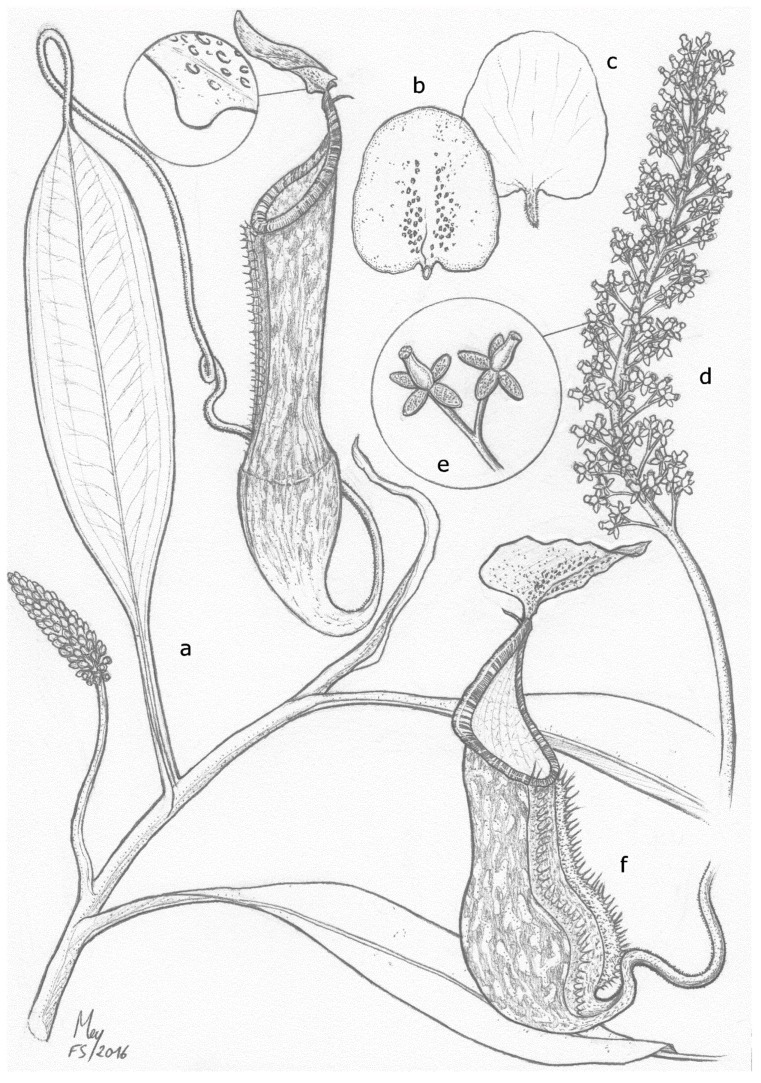
Botanical illustration of *N. justinae.* (**a**) climbing stem with upper pitcher, close up of keel appendage and emerging inflorescence; (**b**) lower side of the upper pitcher lid with glands distribution; (**c**) upper side of upper pitcher lid with unbranched spur; (**d**) female inflorescence; (**e**) partial peduncle with female flowers; (**f**) lower pitcher. Drawing by François Sockhom Mey.

**Figure 4:**
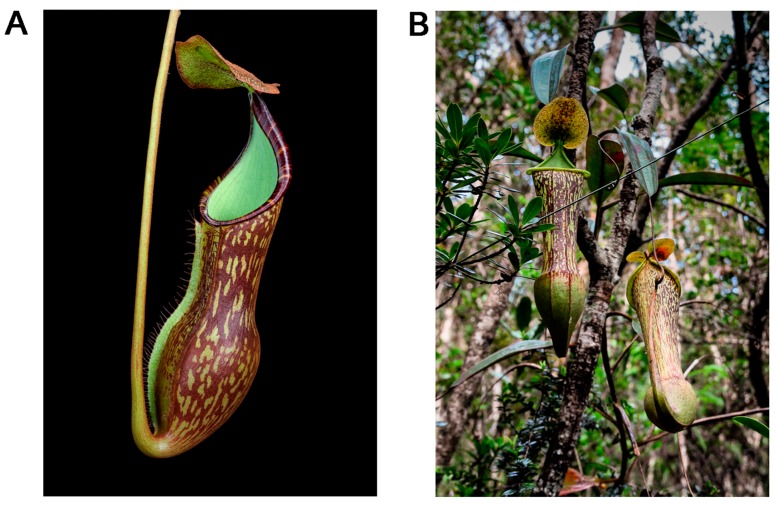
Photographs of *N. justinae*. (**A**) lower pitcher; (**B**) upper pitchers. Photos by Andreas Wistuba and Thomas Gronemeyer

*Nepenthes justinae* is illustrated in Figure 3 and Figure 4.

The morphological features that separate *N. justinae* from *N. mindanaoensis* are summarized in Table 2.

Etymology:

The specific epithet honors Justina Yu, the mayor of the municipality of San Isidro, Davao Oriental Province, Mindanao Island. Due to Justina Yu’s support and dedication, the Mount Hamiguitan Wild Life Sanctuary became a protected national park and was declared a UNESCO World Heritage Site in 2014.

Similarities to other species:

*Nepenthes justinae* belongs morphologically to the *N. alata* group of species. It differs from all of the species of the *N. alata* group in having distinct bulbous lower pitchers. The only other species from the Philippines with bulbous lower pitchers is *N. talaandig* [2]. *Nepenthes justinae* can be distinguished from *N. talaandig* in having lower pitchers lacking a crenellated peristome and in having upper pitchers with a distinct rim that separates the lower ⅓ from the upper ⅔ of the pitcher. The lower pitchers of *N. talaandig* have a crenellated peristome and the upper pitchers lack the distinct rim and are overall more slender than the ones of *N. justinae*. The spur of *N. justinae* is not branched (vs. branched in *N. talaandig*). Furthermore, *N. talaandig* is currently known only from the Pantaron range which is located about 200 kilometres north of Mt. Hamiguitan.

#### 2.2.2. Distribution and Ecology

The species is only known from Mt. Hamiguitan.

*Nepenthes justinae* grows both terrestrially in ultramafic soils and as an epiphyte. The stem usually climbs and scrambles through surrounding vegetation and into the canopy of trees and shrubs. On the Mt. Hamiguitan range, the species grows predominantly in montane forest that covers the summit area of Mt. Hamiguitan (1200 m–1620 m). Scattered specimens have also been recorded growing in ultramafic soils in the pygmy forest at about 1000 m (TG, AW, pers. observation).

*Nepenthes justinae* grows sympatrically with *N. peltata* and *N. micramphora* in the pygmy forest and with *N. hamiguitanensis* in upper montane forest. Hybrids with possible *N. justinae* parentage have been recorded (TG, AW, pers. observation).

#### 2.2.3. Conservation Notes

The Mt. Hamiguitan Range Wild Life Sanctuary was declared a national park in 2003 and a UNESCO World Heritage site in 2014. The national park is a protected area and strong measures have been implemented to prevent unauthorized entrance in the park or poaching. Thus, all *Nepenthes* species of Mt. Hamiguitan are safe from habitat destruction due to surface mining or agriculture for as long as the national park persists and the protective measures are enforced. However, the exact population size of *N. justinae* cannot be estimated since many plants grow epiphytically in tall montane forest trees. As *N. justinae* is endemic to Mt. Hamiguitan, it is assessed here as vulnerable (VU) applying the IUCN Redlist criteria [13].

### 2.3. An Emended Description of Nepenthes ramos Based on Field Data

#### 2.3.1. Emended Taxonomic Description of *N. ramos* and Synonymization of *N. kurata*

***Nepenthes ramos*** Jebb and Cheek
*Nepenthes* *kurata* Jebb and Cheek, European Journal of Taxonomy (2013), 69: 6 *syn. nov.*

Holotype: Philippines, Mindanao Island, April 1919, *Ramos and Palacios s.n.*, K34500 (K)

Material examined:

Philippines, Camiguin Island, Mount Hibok-Hibok, 23.9.2014, *T. Gronemeyer*, CMUH00009782 (CMUH)
Philippines, Mindanao Island, Misamis Occidental province, Mt. Malindang, North Peak, Brgy.
Lake Duminagat, 28.12.2001, *Guiller Opiso and Subanen*, CMUH13153 (CMUH)
Philippines, Mindanao, Surigao del Norte Province, Mt. Masay, 12.5.2015, *T. Gronemeyer s.n.,*
ULM-22541 (ULM) (from a cultivated plant grown from seed)
Material not examined:
Philippines, Mindanao Island, April 1919, *Ramos and Palacios*, K34500 (K; type)
Philippines, Mindanao Island, Mount Malindang, May 1906, *Mearns and Hutchinson 4632* (K)
At the time of writing, these specimens were not made accessible upon request.

*Upper pitchers* are in average 16 cm long and 3–3.5 cm wide at the base and the apex. They are constricted midway to 2.5 cm and sometimes slightly dilated towards the apex. The pitcher opening is up to 3.5 cm wide. Wings run down the front of the trap, fringed with filaments up to 3 mm long. The fringed wings are usually only developed in the upper ⅔ to ¼ of the trap; otherwise wings are reduced to ribs. Wings may also be reduced to ribs throughout. The peristome is 3 mm wide, cylindrical and slightly flattened near the lid. Teeth are barely visible. The lid is ovate to orbicular (broadest at middle), sometimes slightly domed and in average 4 cm wide. In exceptional cases the lid is unusually large and extends the pitcher opening. An appendage of 2 mm on a keel is sometimes present but is mostly reduced to the keel, which is 7–10 mm long and 1–2 mm high. The spur is 9 mm long and unbranched.

The *flowers* have predominantly 2-flowered partial peduncles but on one inflorescence also 1-flowered peduncles can occasionally be found. Fruits are about 2.5 cm long.

The pitcher coloration is very variable; ranging from uniformly pale green over speckled (reddish-brown blotches on green ground) to entirely red.

In all other respects, the observed plants were consistent with the description given by Jebb and Cheek [8].

*Note. Nepenthes ramos* is a very variable species. The dimensions given above are only an average and were taken on representative plants on the volcano Mt. Hibok-Hibok (Camiguin island) that were subsequently cut for the herbarium specimens. Fully functional upper pitchers being only 5 cm long were observed (TG, pers. observation); the largest pitchers measured more than 20 cm. Lower pitchers were not recorded.

The population on Mt. Hibok-Hibok is extensive and comprises dozends if not hundreds of individuals. The habitat is undisturbed since the last eruption of the volcano that took place 65 years ago.

Morphological characteristics to distinguish *N. kurata* as distinct species from *N. ramos* are the lid size and shape, the shape and presence of fringed wings and the pitcher constriction. However, these elements are highly variable in *N. ramos* and thus it is impossible to distinguish *N. kurata* from this species in the field.

**Figure 5:**
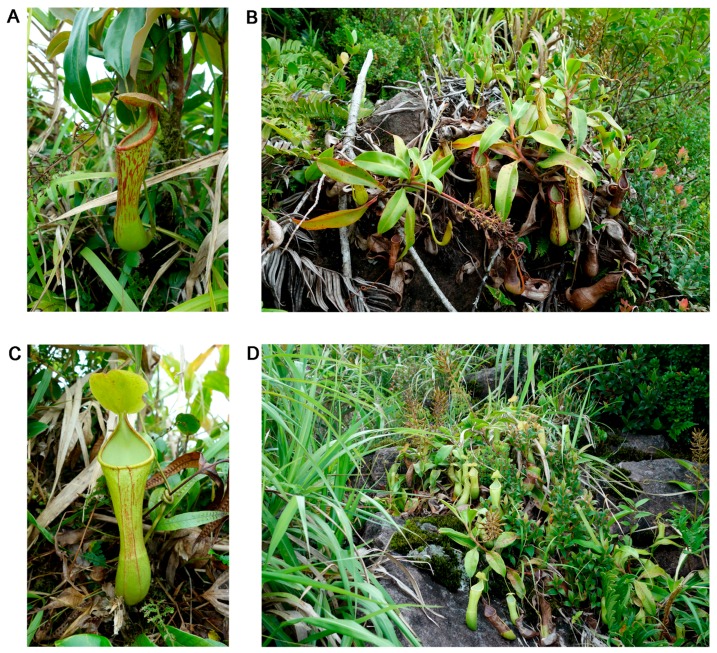
Photographs of *N. ramos* taken on Mt. Hibok-Hibok (Camiguin Island). (**A**, **B**) pitchers with fringed wings; (**C**, **D**) pitchers with reduced or absent fringed wings. Note the variability of the lid size and shape. Photos by Thomas Gronemyer.

Consequently, the two species are synonymized here. The respective relevant diagnostic characters are summarized in Table 3. Figure 5 illustrates the diversity of *N. ramos*. Further photos of *N. ramos* from Mt. Malindang and Mt. Timpoong (Camiguin island) are available online [15].

#### 2.3.2. Ecology and Distribution

*Nepenthes ramos* occupies a wide range of habitats. It can be found in open habitats on volcanic soils as well as in dense forests. The vining stem mostly climbs and scrambles through the surrounding vegetation. Usually the plants grow in dense surrounding vegetation and start to climb at a very early stage. All observed plants grew terrestrially but it is likely that the species is also capable of growing on tree branches. The taxon is widespread on Mindanao and the neighboring islands.

#### 2.3.3. Conservation Notes

The distribution of *N. ramos* is widespread on Mindanao and some adjacent islands. While some habitats are periled by surface mining and agriculture (Diuata Mountains in Surigao del Norte province), others lie in protected areas or national parks (Mt. Hibok-Hibok, Mt. Malindang). It is unlikely that this species will become extinct in the near future but loss of diversity due to habitat destruction is of serious concern. *Nepenthes ramos* is here assessed as “Near Threatened” (NT) under IUCN Redlist criteria [13].

## 3. Discussion

*Nepenthes aenigma* morphologically falls within a Philippine group revolving around *N. ventricosa*, which in turn is included into Danser’s rather loosely defined *Insignes* group, but is unusual in having only three to four longitudinal leaf nerves, and consistently branched fringe elements. Another remarkable trait is the predilection of the intermediate pitchers to be buried in leaf litter, an adaptation commonly interpreted in the genus as a means to more easily trap roving invertebrate prey. This *Nepenthes* is one of the very few species within the genus that is not known to produce lower pitchers.

The accidental discovery of this new species reveals a hitherto unknown adaptation in *Nepenthes*, that is, a propensity to grow in dense shade. *Nepenthes rhombicaulis*, *N. hirsuta* and especially *N. ampullaria* have been noted to grow in shaded localities [12] but the new taxon is unusual in favoring deeply shaded spots overgrown with vegetation. In contrast to all known species within the genus, etiolation due to shade has not been noted, and healthy and large traps are still produced even on situations where light does not appear to penetrate, irrespective of the time of day. The behavior can be interpreted as an adaptation to the very windy and rainy habitat where it grows, but its ecological preferences appear to be the most extreme known so far in any *Nepenthes*. This raises the odds that other *Nepenthes* with similar ecological predilection may still be found elsewhere.

*Nepenthes aenigma* is the third *Nepenthes* to have been described from the province of Ilocos Norte, after *N. alata* and *N. ventricosa* were described by Spanish friar Manuel Blanco in 1837 from plants found in Vintar.

Similar to *N. aenigma*, the herein newly described *N. justinae* has a very narrow distribution. However, *N. justinae* morphologically belongs to the large *N. alata* group of species. *Nepenthes justinae* is the fourth endemic *Nepenthes* of Mt. Hamiguitan beside *N. peltata*, *N. micramphora* and *N. hamiguitanensis*. This finding decorates Mt. Hamiguitan with the highest number of single-mountain endemic pitcher plants.

Both *N. aenigma* and *N. justinae* are described in this work based on field data that should allow for a reliable identification of these species in the field.

Many other species described recently were differentiated from each other only by minor characteristics and often only based on few if not only one (dried) specimen [3,4,5,6,8,9,10]. However, a certain degree of variability can always been recorded when observing pitcher plants in their natural habitat and hybridization is always a concern. *Nepenthes extincta* (Surigao del Norte province, Mindanao Island) may serve as an example for this concern: The species was described based on a single incomplete herbarium specimen that was found on ultramafic soils near the municipality of Claver [10]. The holotype contains only intermediate pitchers and no flowers. According to the species description, *N. extincta* differs from *N. mindanaoensis* in “the pitchers lacking fringed wings, the lid base truncate, the indumentum of the midrib of dense minute grey-white stellate hairs“ [10]. *Nepenthes mindanaoensis* grows sympatrically with *N. merrilliana* (the latter having upper pitchers lacking fringed wings) on ultramafic soils on the adjacent Dinagat Island. The two species hybridize freely (TG, AW, pers. observation). As the latter two species are known to grow also in the vicinity of the *N. extincta* *locus classicus* (Stewart McPherson, pers. communication) in NE Mindanao, one cannot exclude that the single known *N. extincta* specimen represents a *N. merrilliana x mindanaoensis* hybrid.

*Nepenthes ramos* was described based on herbarium specimens of adult plants including flowers [8]. However, this material is only composed of a few individual adult plants. Our emended description underscores that the observation of only few individuals may not be sufficient to cover the full diversity of a taxon. Instead, the growth habit and ecology of each species provide useful characters that should also be considered. For example, on Mount Hibok-Hibok (Camiguin island), a nice diversification pattern of *N. ramos* can be observed that contradicts some of the known “stable” characters that were used in the past to separate *Nepenthes* taxa from each other.

Mount Hibok-Hibok is an active volcano; the last major eruption dates back to 1951. In this pelean eruption, the whole crater was completely destroyed and the slopes of the mountain were devastated by lava- and pyroclastic flows and landslides. Vegetation settled again in the years after, suggesting that the whole population of *N. ramos* in the Mt. Hibok-Hibok crater originates from a few seeds that were transported to the mountain.

If several individual dried specimens from Mt. Hibok-Hibok would have been considered for species description, one could easily separate each of these into a distinct species on its own. Only careful study of the plants *in situ* and considering the ecological history of the mountain revealed that only one single species exhibiting a great variability is present on Mt. Hibok-Hibok.

The characteristics that separate *N. kurata* from *N. ramos* are minor and fall in the variability of the latter species. Maybe other closely related species from the Visayas that have not yet been studied in the field (like *N. leyte* or *N. negros)* likewise represent *N. ramos.* However, as the authors did not conduct field research on the Visayas, *N. leyte* and *N. negros* are retained here as distinct species.

## 4. Material and Methods 

Field research in Ilocos Norte Province, Luzon, was conducted in April 2002 (HN), June 2007 (MC), June 2011 (MC), April 2013 (WS) and May 2015 (HN, WS, MC).

Field research at Mt. Hamiguitan was carried out during August 2004 (VBA), July 2008 (TG) and January 2010 (TG, AW).

Field research on Dinagat Island was carried out during September 2007 (TG), January 2010 (AW, TG) and August 2012 (AW).

Field observations of *N. ramos* were made at the following locations:

Mt. Hibok-Hibok, Camiguin Island (September 2007, August 2011 and September 2014) (TG)
Mt. Masay, Surigao del Norte Province, Mindanao (September 2007 and July 2008) (TG)
Pantaron Range, San Fernando area, Bukidnon Province, Mindanao (July 2008) (TG)
Mt. Malindang, Misamis Occidental Province, Mindanao (December 2001) (VBA)

Plant material was collected under permits granted to MC and VBA.

Cropped plant material was processed using standard methods at the herbarium of the Central Mindanao University in Valencia, the herbarium of North-Western University in Laoag and the herbarium of the Botanical Garden Ulm.

Photographs were made from suitable, representative plant specimens *in situ* or from cultivated plants grown from seed. Measurements were taken using a graded ruler or a digital caliper.

## 5. Conclusions 

Two new taxa of carnivorous pitcher plants, *N. aenigma* and *N. justinae* are presented and formally described here. Furthermore, an emended description of *N. ramos* based on field data is provided which includes the synonymization of *N. kurata*.

In the discussion, the authors point out the urgent need of solid data recorded in the field for the description of new taxa, especially when only minor characters are used for the separation of species.

## Figures and Tables

**Table 1:** Major characteristics separating *N. aenigma* from *N. ventricosa* and *N. burkei*. Data of *N. ventricosa* *and N. burkei* were taken from [12].

	*Nepenthes ventricosa*	*Nepenthes burkei*	*Nepenthes aenigma*
Growing habit and habitat	Terrestrial, epiphytic or lithophytic. In bright to shady conditions.	Terrestrial or epiphytic. In bright to shady conditions.	Terrestrial in deep shade.
Lamina	Long and narrow, length-breadth ratio ±6.4:1	Long and narrow, length-breadth ratio ±6:1	Shorter and broader, length-breadth ratio ±3.5:1
Lower pitchers	Absent on mature plants	Absent on mature plants	Absent on mature plants
Comparison intermediate and upper pitchers	Monomorphic	Monomorphic	Strongly dimorphic
Pitcher shape	Strongly narrowed in the middle, forming a distinct waist.	Slightly narrowed in the middle or cylindrical.	Cylindrical with widely infundibular base in upper pitcher
Pitcher opening	Horizontal to slightly oblique	Strongly oblique	Intermediate between *N. ventricosa* and *N. burkei*
Wings	Reduced to ridges or not apparent	Reduced to ridges or not apparent	Prominent on all pitchers
Peristome	Up to 2 cm wide with teeth up to 1 mm long	Up to 3 cm wide with teeth up to 2 mm long	Less than 1 cm wide with teeth less than 1 mm long
Inflorescence	1-flowered partial peduncles	1-flowered partial peduncles	2-flowered partial peduncles

**Table 2:** Major characteristics separating *N. justinae* from *N. mindanaoensis*. Data from *N. mindanoensis* were taken from [14].

	*Nepenthes justinae*	*Nepenthes mindanaoensis*
Lower pitchers	Bulbous in the lower ⅔, slightly infundibulate in the upper ⅓.	Obovate or subglobose in the lower ¼, otherwise cylindrical
Upper pitchers	Strongly dimorphic	Overall shape similar than the lower pitchers
Pitcher opening	Strongly oblique (lower pitcher)	Slightly oblique
Nectar glands	Monomorphic	Dimorphic
Appendage	Present in upper pitchers	Absent

**Table 3:** Diagnostic characteristics of *N. ramos*, *N. kurata* and *N. mindanaoensis*. Note that all characteristics of *N. kurata* correspond with those of *N. ramos*.

	*Nepenthes ramos emend.*	*Nepenthes kurata* [10]	*Nepenthes mindanaoensis* [14]
Upper pitchers	Mostly equally wide at base and apex, sometimes slightly dilated towards the apex. Constricted in the middle to various extent.	Slightly dilated towards the apex. Constricted in the middle.	Obovate or subglobose in the lower ¼, otherwise cylindrical.
Fringed wings	Present in the upper ⅔ to ¼ of the trap or reduced to ribs.	Present in the upper ⅓ to ¼ of the trap.	Present on the whole length of the pitcher.
Lid	Smaller, as large as or larger than the pitcher opening. Ovate to orbicular, sometimes domed.	Smaller than the pitcher opening. Ovate.	About as large as the pitcher opening. Orbicular.
Nectar glands	Dimorphic	Dimorphic	Dimorphic
Appendage	Present (2 mm) or reduced to keel.	Modestly developed, 1–2 mm	Absent

## References

[1] Robinson A.S., Fleischmann A.S., Mcpherson S.R., Heinrich V.B., Gironella E.P., Peña C.Q. (2009). A spectacular new species of *Nepenthes* L. (Nepenthaceae) pitcher plant from central Palawan, Philippines. Bot. J. Linn. Soc..

[2] Gronemeyer T., Coritico F., Wistuba A., Marwinski D., Gieray T., Micheler M., Mey F.S., Amoroso V. (2014). Four new species of *Nepenthes* L. (Nepenthaceae) from the central mountains of Mindanao, Philippines. Plants.

[3] Cheek M., Jebb M. (2013). Typification and redelimitation of Nepenthes alata with notes on the N. alata group, and N. negros sp. nov. from the Philippines. Nordic J. Bot..

[4] Cheek M., Jebb M. (2013). Nepenthes alzapan (Nepenthaceae), a new species from Luzon, Philippines. Phytotaxa.

[5] Cheek M., Jebb M. (2013). *Nepenthes ultra* (*Nepenthaceae.*), a new species from Luzon, Philippines. Blumea.

[6] Cheek M., Jebb M. (2013). Identification and typification of Nepenthes blancoi, with *N. abalata* sp. nov. from the western Visayas, Philippines. Nordic J. Bot..

[7] Cheek M., Tandang D.M., Pelser P.B. (2015). Nepenthes barcelonae (Nepenthaceae), a new species from Luzon, Philippines. Phytotaxa.

[8] Cheek M., Jebb M. (2013). *Nepenthes ramos* (Nepenthaceae), a new species from Mindanao, Philippines. Willdenowia.

[9] Cheek M., Jebb M. (2013). The *Nepenthes micramphora* (Nepenthaceae) group, with two new species from Mindanao, Philippines. Phytotaxa.

[10] Cheek M., Jebb M. (2013). Recircumscription of the Nepenthes alata group (Caryophyllales: Nepenthaceae), in the Philippines, with four new species. Eur. J. Taxon..

[11] McPherson S. (2012). New Nepenthes Vol. 1.

[12] McPherson S. (2009). Pitcher Plants of the Old World.

[13] IUCN The IUCN Red List of Threatened Species. http://www.iucnredlist.org.

[14] Kurata S. (2001). Two new species of Nepenthes from Sumatra (Indonesia) and Mindanao (Philippines). J. Insectivorous Plant. Soc..

[15] Co’s Digital Flora of the Philippines. https://www.facebook.com/groups/philippineplants/.

